# Soil selenium and oesophageal cancer incidence in China: a large ecological study of population-based cancer registry data

**DOI:** 10.1186/s12942-026-00452-y

**Published:** 2026-02-06

**Authors:** Shuanghua Xie, Xianhui Ran, Liacine Bouaoun, Gerrad Jones, Christian Abnet, Anthony Kityo, Wenqiang Wei, Valerie McCormack, Daniel R. S. Middleton

**Affiliations:** 1 Central Laboratory Department, Beijing Obstetrics and Gynaecology Hospital, Capital Medical University, Beijing Maternal and Child Health Care Hospital, Beijing, China; 2 National Central Cancer Registry, National Cancer Centre/National Clinical Research Centre for Cancer/Cancer Hospital, Chinese Academy of Medical Sciences and Peking Union Medical College, Beijing, China; 3 Environment and Lifestyle Epidemiology Branch, International Agency for Research On Cancer, Lyon, France; 4 College of Agricultural Sciences, Department of Biological & Ecological Engineering, Oregon State University, Corvallis, OR USA; 5 National Cancer Institute, Rockville, MD USA; 6 Centre for Public Health, Queen’s University Belfast, Belfast, UK

## Abstract

Incidence rates of oesophageal cancer (EC), predominantly oesophageal squamous cell carcinoma (ESCC), in areas of China are the highest worldwide. Selenium, a trace element linked to ESCC risk, likely plays a role in ESCC’s enigmatic spatial distribution. We investigated the association between soil selenium and EC incidence in China. We conducted a large ecological study using 2016 population-based EC incidence data from 486 cancer registry catchments covering 380 million people and 74,000 EC cases. We assigned mean soil selenium concentrations to each area from geospatial maps. Age-standardized EC incidence rates (ASRs) were computed. We used linear regression models to estimate approximate incidence rate ratios (IRRs) for ASRs across soil selenium quintiles and for areas classified as deficient (≤ 0.2 mg/kg). The distribution of ASRs differed above and below the selenium deficiency threshold (0.2 mg/kg). Above, 100% of ASRs in females and 87% in males were < 15/100,000. Below, 81% of ASRs in females and 36% in males were < 15/100,000, with ASRs having a wide range (0 – 117.5 per 100,000 person-years). Soil selenium-deficient areas were linked to more than twofold increased EC incidence among males (IRR: 2.45; 95% CI: 2.13, 2.81) and threefold among females (IRR: 3.35; 95% CI: 2.67, 4.19). These findings support the hypothesis of selenium’s role in the incidence of EC, which may arise from increased susceptibility to the carcinogenic effects of other exposures in selenium-deficient areas. In China, all EC hotspots occur in selenium deficient areas, yet there are selenium deficient areas with low EC rates.

## Introduction

Oesophageal cancer (EC) incidence and mortality rank 6th among all cancers worldwide, with an estimated 511,054 new cases and 445,391 deaths globally in 2022 [1]. These figures highlight the high public health burden and dire prognosis of the disease, which is often diagnosed too late for curative treatment. Primary prevention through risk factor identification and early detection of EC are the most promising avenues to reducing its burden at present.

Predominating as two histological subtypes, EC diagnoses in ‘Western’ countries are more often adenocarcinomas (EAC), which have seen a steady increase in incidence over the last three decades, particularly in males, and for which gastrointestinal reflux disease is a strong risk factor [2]. Oesophageal squamous cell carcinoma (ESCC) is the dominant subtype in most of the world and displays some of the most distinctive geographical contrasts in incidence rate than any other cancer type. Notable high ESCC incidence ‘hotspots’ include the extensively documented Asian ESCC belt including Iran and China [3], and the easterly lying African ESCC corridor, spanning from Ethiopia to South Africa’s Eastern Cape [4].

Risk factors for ESCC described in lower incidence Western settings, such as excessive alcohol consumption and tobacco smoking, also play an appreciable aetiological role in some but not all high incidence areas [5, 6], with evidence of the carcinogenic effects of very hot beverage consumption [7, 8], and household air pollution [9] also emerging. Nevertheless, none of these environmental and lifestyle factors fully demarcate observed regional differences in ESCC incidence. For example, in China, which has some of the highest localised ESCC incidence in the world, rates between areas can differ by up to three orders of magnitude – with age-standardized incidence rates (ASR) exceeding 100 per 100,000 person-years in the most affected communities [10]. Such differences are therefore unlikely explained solely by lifestyle factors.

These observations have led some authors to hypothesize a role of spatially patterned environmental variables in ESCC aetiology. In particular, the detrimental effects of deficiencies in geochemically mediated soil trace elements have long been postulated [11, 12]. The latter perspectives built on the observations that the micronutrient selenium is a common factor displaying primary deficiencies in both the high ESCC risk Chinese and African hotspots. Keshan disease (a cardiomyopathy associated with selenium deficiency) was also prevalent in the Chinese high-risk areas. Selenium, as well as being toxic at high doses, is a component of selenoproteins and selenoenzymes involved in the protection against numerous carcinogenic mechanisms [13] such as DNA repair [14], antioxidation [15], anti-inflammation [16], and immunity [17]. Epidemiological evidence is heterogenous, with some cross-sectional and ecological studies reporting modest positive (i.e. detrimental) associations between selenium and oesophageal cancer [18], or its precursor oesophageal squamous dysplasia [19]. Furthermore, regarding cancers at other anatomical sites, no beneficial effect of selenium supplementation was found in a randomized control trial of non-small cell lung cancer [20], and an increased cancer risk was detected in individuals with higher selenium status in a randomized control trial of prostate cancer [21]. Other prospective designs support a protective role of selenium against ESCC. For example, in a prospective case-cohort study of participants in the Linxian General Population Trial in China, a strong negative association between baseline serum selenium and risk of EC was found, with a relative risk (RR) of 0.56 (95% CI: 0.44, 0.71) reported [22]. In the same cohort, a RR of 0.83 (95% CI: 0.71, 0.98) was reported per 25% increase in serum selenium after 15 years of follow-up [23]. Similarly, in the prospective Netherlands Cohort Study, selenium measured in toenail clippings collected at baseline was also inversely associated (RR per standard increment: 0.80, 95% CI: 0.67, 0.96) with ESCC risk specifically, and not with EAC [24].

The possibility of the chemopreventive action of selenium has also been demonstrated by a randomized controlled trial [25] of a 200 µg/day dose of selenomethionine, which resulted in oesophageal squamous dysplasia regression, and inhibited progression among low grade, but not moderate grade, dysplasia patients in China.

**Fig. 1:**
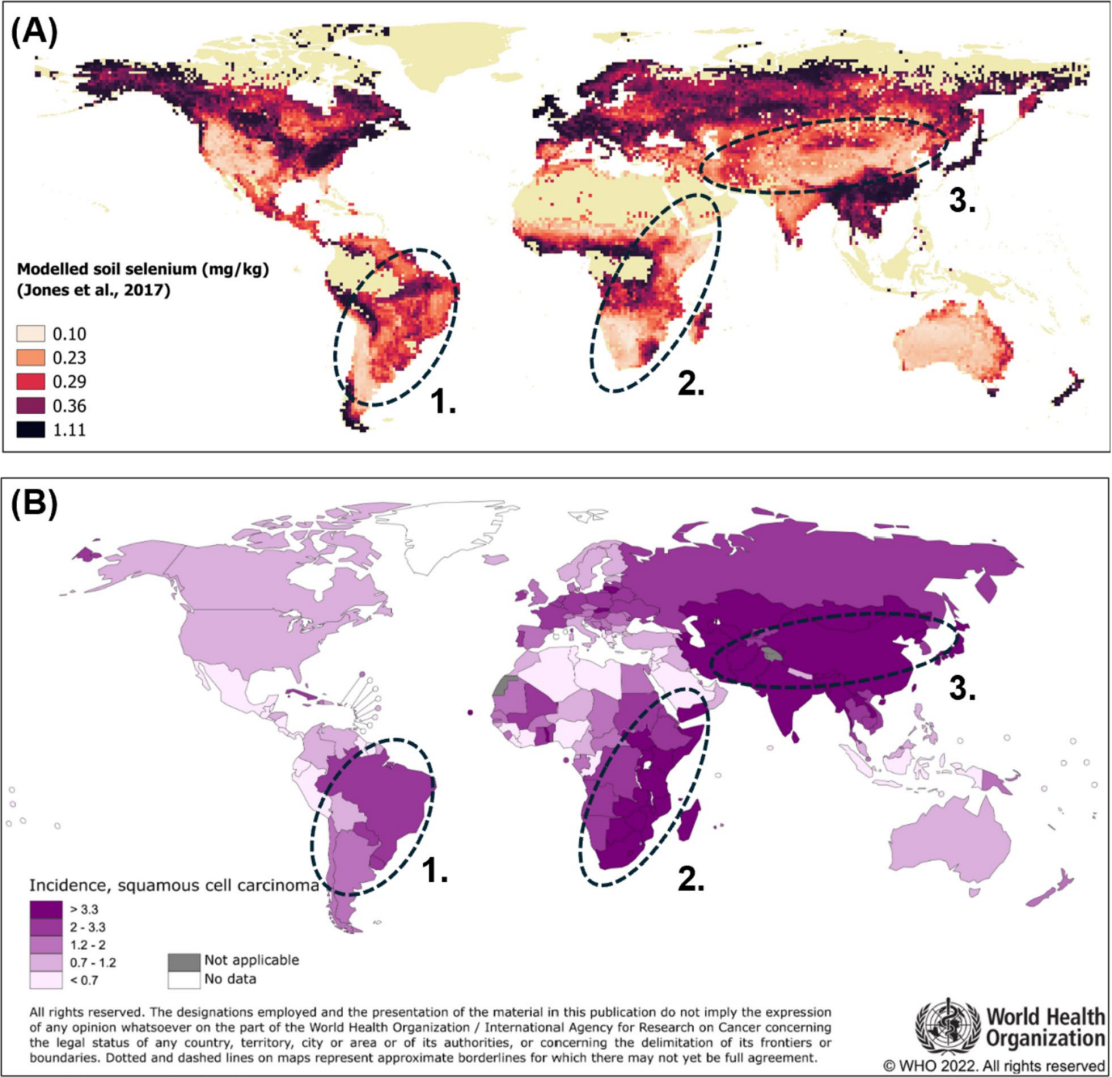
**(A)** Modelled soil selenium concentration [26] and **(B)** oesophageal squamous cell carcinoma (ESCC) incidence [27] worldwide, showing an apparent overlap of low selenium and high-ESCC regions of: 1. South America; 2. East Africa; and 3. Asia

The aetiology of ESCC is evidently multifactorial, involving the carcinogenic effects of long since established carcinogens such as tobacco, alcohol and yet unidentified environmental exposures. While there is compelling evidence for a protective role of selenium, its contribution to the puzzling spatial distribution of ESCC incidence remains unclear. Visual inspection of maps such as those shown in Fig. 1 reveals an apparent collocation of known ESCC hotspots and regions of lower predicted soil selenium concentrations. However, conducting ecological studies at the global scale is challenged by the vast socio-demographic, environmental, and cultural differences between world populations and their potential to confound associations, as well as a lack of high-quality population-based cancer registry statistics in some of the most affected low- and middle-income countries (LMICs) such as those in East Africa. One such study did however find an ecological association between food balance sheet selenium intake estimates and country-specific (GLOBOCAN 2012) EC incidence rates, reporting an incidence rate ratio of 0.40 (95% CI: 0.18, 0.90) [12]. Despite the design’s limitations, an ecological study conducted on a sufficiently large number of aggregate units, within the confines of a single high-ESCC incidence country which has seen a substantial increase in high-quality population-based cancer registries, and whose rural population still relies heavily on subsistence crops, may provide further insights into an already strong hypothesis of selenium intake and reduced ESCC risk.

In this large population-based ecological study, we aimed to investigate the association between soil total selenium and EC incidence rates in China—a country with high EC incidence, and areas of well-documented endemic selenium deficiency in the population [28].

## Methods

### Study design

Overall, the study design was an ecological association study between the average soil selenium concentrations and EC incidence rates, where the ecological observational unit was the catchment population of each contributing population-based cancer registry (PBCR).

### Cancer incidence data

Data on incidence rates of EC (ICD-10: C15) in China were extracted from the 2019 China Cancer Registry Annual Report published by the National Cancer Centre [29], which reports cancer cases diagnosed in the 2016 calendar year following data review and quality control. These data were collected and reported according to the Chinese Cancer Registration Guidelines 2016 [30] and the standards of the International Agency for Research on Cancer/International Association of Cancer Registries (IARC/IACR) [31]. The 2019 Annual Report included a total of 486 individual PBCRs following data review and quality control. Each PBCR serves a catchment area, for which boundaries are specified and were made available, as well as the population size covered by each area. All catchment areas combined covered a population of 382 million, accounting for 27.6% of the national Chinese population as of the end of 2016. Age-specific incidence rates were calculated using the corresponding PBCR catchment population denominators for 2016, stratified by age and sex. Age-standardized incidence rates (ASR, expressed per 100,000 person-years) were estimated separately for males and females, using the corresponding population age composition of the fifth national population census from the year 2000 as the standard population, as the reference.

### Soil total selenium data

A China-wide map of total soil selenium concentration was prepared in a previous study [32] using measurement data from China’s Soil Environment Background Concentration Research [33] and other studies providing data on soil trace element background values in China [34, 35]. Note that soil selenium concentration is relatively constant in the medium to long term.

### Mapping of area-level exposure and outcome

**Fig. 2:**
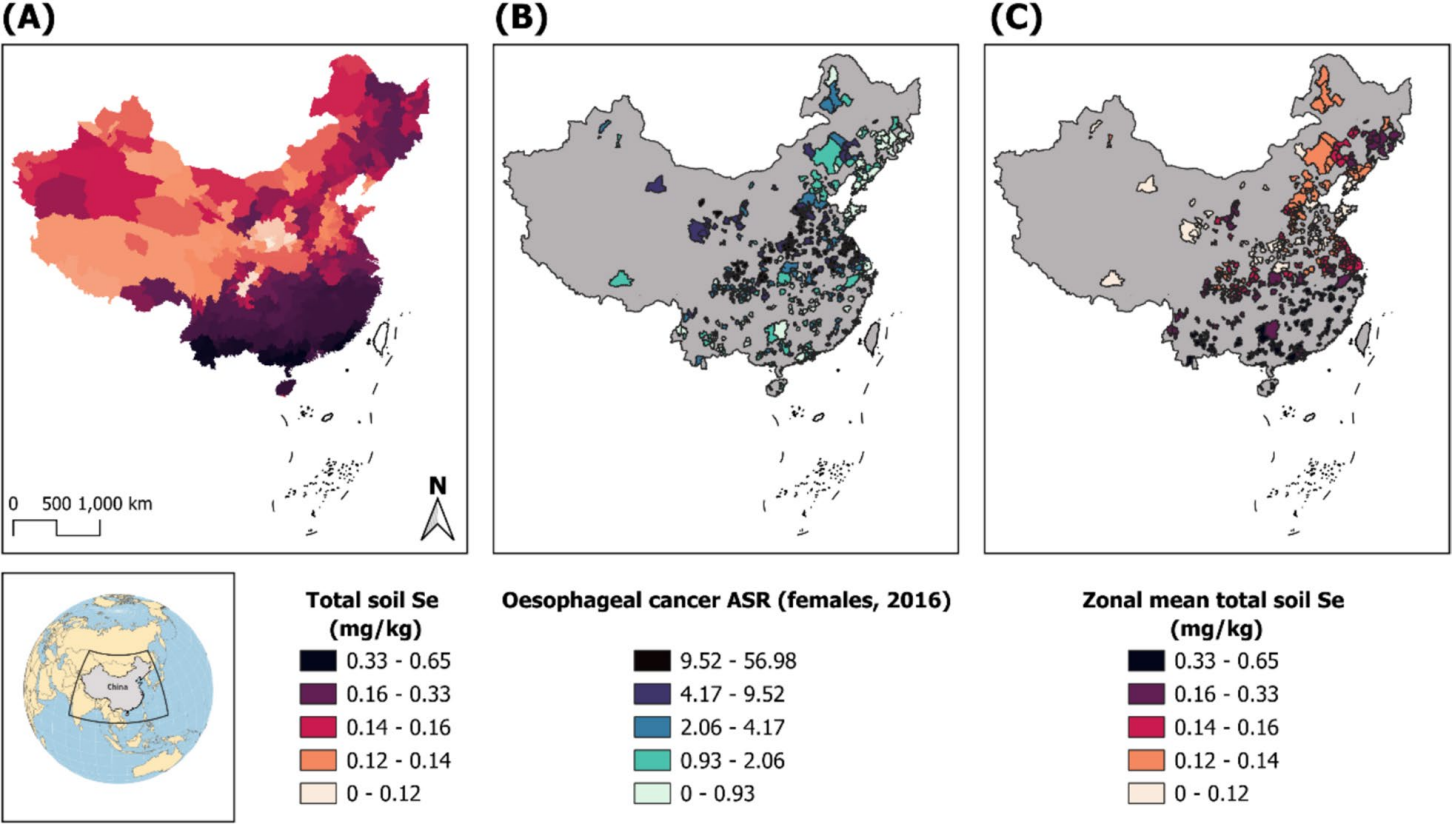
Area-level aggregated soil selenium ascertainment by **(A)** rasterization of a prefecture-level soil selenium map [32],**(B)** mapping 486 cancer registry catchment area boundaries (female oesophageal cancer age-standardized incidence is plotted for illustration); and **(C)** extracting underlying soil selenium zonal means for each cancer registry boundary. Grey (in A,B) denotes areas not covered by population-based cancer registries

First, PBCR catchment area boundaries were prepared from shapefiles downloaded from the Database of Global Administrative Areas (GADM) in a Geographic Information System (GIS) using the Quantum GIS software package (QGIS version 3.30.2). PBCR data were reported at Level 3, which divides the country into 2,854 counties (*Xiàn*), of which 486 correspond to the aforementioned PBCRs. Second, a shapefile of the soil total selenium map [32] aggregated at the prefecture division level (339 divisions) was imported into GIS and converted into a raster image at a 1 km grid-square resolution to generate a continuous sampling space. A zonal statistics geoprocessing tool was then used to calculate the 486 PBCR catchment area-level means of selenium raster cell values (mg/kg) within the confines of each of boundary. As noted, selenium data were available only at the prefecture level (*n* = 339) from secondary sources, whereas cancer incidence data were reported primarily at county level (*n* = 486). Consequently, approximately 10% of PBCRs within the same prefecture were assigned identical selenium values. Rasterization of the selenium map to a 1-km grid was undertaken solely to enable GIS overlay and does not increase resolution. This partial spatial mismatch is expected to result in non-differential exposure misclassification and therefore bias effect estimates toward the null rather than generate spurious associations. This process and the units of analysis are depicted in Fig. 2. Note that because PBCR areas (most at county-level) are typically much smaller than the selenium map resolution (at prefecture level, of which there are 8 times as many counties as prefectures), neighbouring cancer registries within the same prefecture will have the same selenium levels.

### Statistical analysis

Primarily, the associations between soil selenium and EC ASRs in China were inspected visually using scatterplots and quantified using Spearman’s rank correlation coefficients, for males and females separately. The association between mean soil selenium and EC incidence was also quantified using linear regression models to calculate approximate Incidence Rate Ratios (IRRs) and their 95% confidence intervals (95% CIs). ASRs were log-transformed prior to regression analyses. Two separate linear models were considered, based on the soil selenium exposure variable. A first model (*Model 1*) used soil selenium in quintiles (Q1: < 0.12 mg/kg; Q2: 0.12—0.14 mg/kg; Q3: 0.14—0.16 mg/kg; Q4: 0.16 – 0.32 mg/kg; Q5 > 0.32 mg/kg), with IRRs computed for all quintiles and Q5 set as the reference category. A second model (*Model 2*) was used to investigate the EC rates in relation to living in a catchment population with soil selenium concentration of ≤ 0.2 mg/kg – a cutoff previously used to define soils low/deficient in selenium in a study of the element’s distribution in Chinese soils [36] – as a binary variable. Across both models, IRRs were calculated separately by sex. All statistical analyses were conducted using the R programming language (version 4.3.1) [37] with R Studio. *P* values are two sided and a significance level was set to 0.05.

## Results

Across 486 PBCR areas included in the analysis, EC ASRs ranged from 0 to 117.5/100,000 person-years in males and 0 to 57/100,000 person-years in females. Soil selenium concentrations ranged from 0.08 – 0.65 mg/kg. Counties with the highest male-specific EC ASRs per 100,000 person-years were Ci Xian (117.5), Yan Ting Xian (91.8), Yang Cheng Xian (89.0), Luo Ning Xian (82.9), and Lang Zhong Shi (82.8). All these areas had mean soil selenium concentrations below 0.15 mg/kg, which, in the absence of a definitive cut-off, has been considered low/deficient in previous studies [36]. The same areas also accounted for the highest female-specific rates, but 40% lower than in males—Luo Ning Xian (57.0), Ci Xian (55.7), Yan Ting Xian (50.9), Lang Zhong Shi (49.2), and Yang Cheng Xian (47.6). The counties with the lowest male-specific rates were Ma Yang Miao Zu Zi Zhi Xian (0.0) – though we note zero recorded cases may reflect registration error, Yu Xi Shi Jiang Chuan Qu (0.81), Shang Gao Xian (0.85), Jing An Xian (1.8), and Zhu Zhou Shi Lu Song Qu (2.08), all having mean soil selenium concentrations above 0.25 mg/kg, and four out of five surpassing 0.35 mg/kg. For females, the lowest ASRs were found in Qian An Xian, Nan Chang Shi Wan Li Qu, Shang Gao Xian, Heng Feng Xian, and Zhu Zhou Shi Shi Feng Qu – all reporting zero cases of EC in 2016, and all but one (Qian An Xian—0.15 mg/kg) with mean soil selenium concentrations above 0.3 mg/kg.

**Fig. 3:**
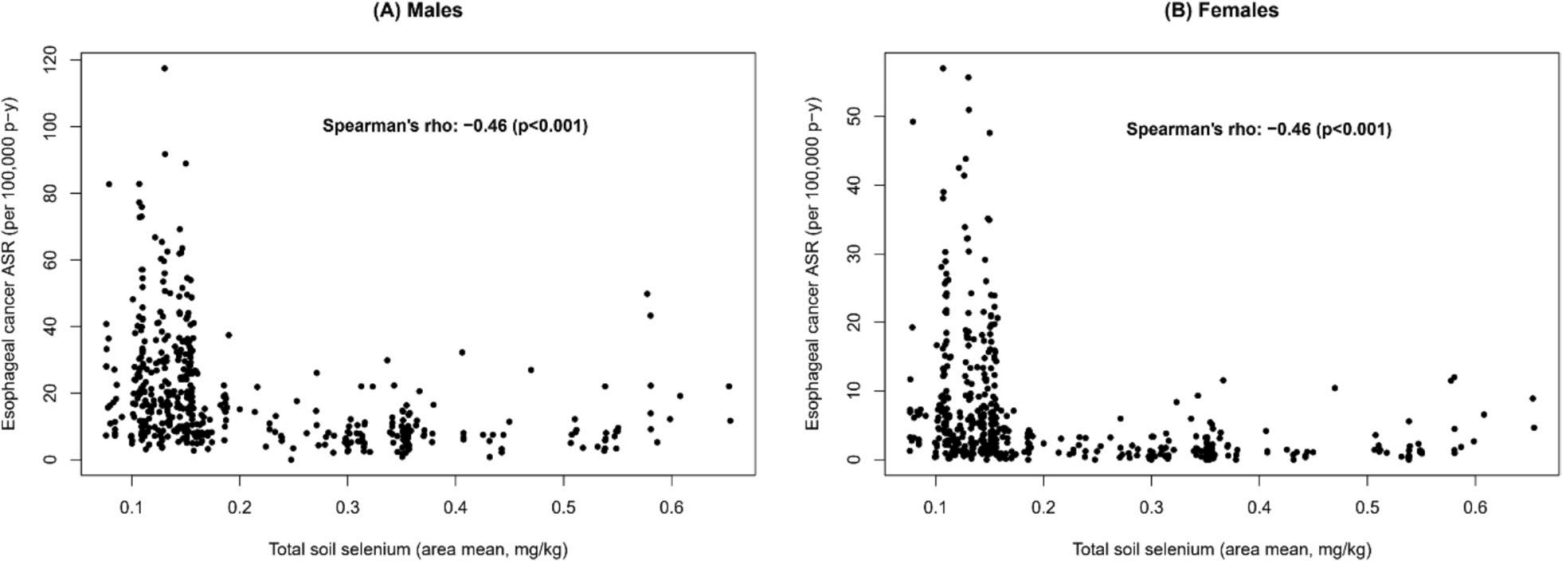
Scatterplots and Spearman’s correlation coefficients illustrating age-standardized oesophageal cancer incidence plotted against area-level (*n* = 486) soil selenium among **(A)** males, and **(B)** females

Scatterplots and Spearman’s rank correlation coefficients of EC incidence against mean soil selenium concentration are shown for males and females separately in Fig. 3. Visual inspection of these plots reveals an L-shaped association, whereby incidence rates are generally low in areas with soil selenium concentrations above 0.15 mg/kg.

Notably, whereas EC ASRs were mostly low (< 10 in females and < 20 in males) in areas where selenium was adequate (> 0.2 mg/kg), in areas where selenium was inadequate, EC ASRs spanned from very low to extremely high rates. Two identical and statistically significant Spearman’s correlation coefficients were calculated for males and females (-0.46, *p* < 0.001), showing a moderate negative correlation between soil selenium and EC incidence.

The IRRs for EC associated with mean soil selenium levels are shown in Table 1. In *Model 1*, there were higher EC incidence rates for selenium concentrations below 0.16 mg/kg, with male-specific IRRs of 2.56 (95% CI: 2.13, 3.09), 2.72 (95% CI: 2.20, 3.36), 2.58 (95% CI: 2.13, 3.13) for Q3, Q2, and Q1 respectively, compared to Q5. Similar results were observed among females with IRRs of 3.33 (95% CI: 2.47, 4.49), 3.43 (95% CI: 2.44, 4.81), 3.53 (95% CI: 2.60, 4.80) for Q3, Q2, and Q1. In *Model 2*, IRRs were estimated for areas with mean soil selenium concentrations in the range classified as deficient or low (≤ 0.2 mg/kg). Statistically significant elevated IRRs were observed in both males (2.45; 95% CI: 2.13, 2.81) and females (3.35; 95% CI: 2.67, 4.19), when comparing areas with deficient or low (≤ 0.2 mg/kg) mean soil selenium concentrations to areas with > 0.2 mg/kg.

**Table 1:** Sex-specific approximated Incidence Rate Ratios (IRR) and 95% confidence intervals (95% CIs) showing the association between area-level soil selenium concentration and oesophageal cancer incidence rates

Group^a^	Exposure category	IRR (95% CI)
*Model 1 – soil selenium quintiles (mg/kg)*		
Males (*n* = 484)	Q5: > 0.32 (reference) Q4: 0.16—0.32 Q3: 0.14—0.16 Q2: 0.12—0.14 Q1: < 0.12	1 (ref) 1.15 (0.94, 1.41) 2.56 (2.13, 3.09) 2.72 (2.20, 3.36) 2.58 (2.13, 3.13)
Females (*n* = 475)	Q5: > 0.32 (reference) Q4: 0.16—0.32 Q3: 0.14—0.16 Q2: 0.12—0.14 Q1: < 0.12	1 (ref) 0.86 (0.62, 1.19) 3.33 (2.47, 4.49) 3.43 (2.44, 4.81) 3.53 (2.60, 4.80)
*Model 2 – ‘deficient/low’ soil selenium*		
Males	> 0.2 mg/kg (reference)	1 (ref)
(*n* = 485)	≤ 0.2 mg/kg	2.45 (2.13, 2.81)
Females	> 0.2 mg/kg (reference)	1 (ref)
(*n* = 476)	≤ 0.2 mg/kg	3.35 (2.67, 4.19)

^a^Observation numbers for each model differ slightly from one another and from the correlation analyses due to the omission of a small number of areas with zero case counts. “(ref)”—reference category. Male and female-specific effect estimates are intended for within-sex interpretation, with comparisons across sexes limited to the qualitative presence and direction of associations rather than formal comparison of effect sizes

## Discussion

Our study investigated the ecological association between soil selenium concentration and EC incidence rates in China. To our knowledge, this study represents the largest ecological analysis of soil selenium and EC to date, incorporating 486 units of analysis representing a population of over 380 million people and more than 74,000 EC cases within a single country, including geographical units where the EC rates are the highest worldwide and units with 0 cases reported in a year. We found a statistically significant negative association between soil selenium concentrations and EC incidence. Areas with soil selenium deficiency had a more than twofold male-specific EC incidence and more than threefold female-specific EC incidence compared to areas with adequate soil selenium.

The observed association was L-shaped, with a wide range of EC incidence rates in selenium deficient areas. Variations in incidence at low selenium levels therefore appear to be driven by other factors, whose carcinogenic effects may be offset by selenium at higher levels of supply, assuming that our reported association is causal in nature. Interaction between other EC risk factors and low selenium levels may explain the large range of EC incidence rates within the low selenium geographies. This interpretation supports the hypothesis of a deterministic role of selenium in the spatial distribution of EC while still aligning with the substantial EC burden attributable to lifestyle risk factors [5] observed in high-incidence settings.

Our findings are tempered by several notable limitations. Due to its ecological design, this study is inherently susceptible to ecological fallacy, meaning the area-level associations presented may not represent individual-level associations in the underlying population. However, the availability of existing individual-level observational data from more robust epidemiological study designs on selenium status and EC risk [22, 23] in this and other settings [24] adds consistency to our findings. The study’s reliance on routine data also makes it vulnerable to any underlying limitations regarding data accuracy and validity. While both cancer registration and soil selenium data were compiled from trustworthy sources, uncertainty remains regarding the representativeness of fixed PBCR catchment area boundaries, which do not account for population migration that may have occurred during the cancer’s long latency period. Nevertheless, this effect may balance out given both the large number of units in the analysis and the substantial population under study.

Another limitation arises from the disconnect between true human selenium status and area-level estimates of soil selenium, with the latter assumed to be a suitable proxy for the former. This assumption is supported to some extent by previous investigations into the geographic distribution of soil selenium [36] and human selenium status, health, and disease in China [38]. For example, Keshan disease—an endemic cardiomyopathy partially caused by dietary selenium deficiency—occurs in a north-easterly running belt from Yunnan Province to Heilongjiang Province [39], the same belt characterized by registry catchments with low estimated soil selenium and high EC incidence in our analysis (Fig. 2B). Furthermore, despite ongoing dietary transitions, a high proportion of China’s rural population still relies on subsistence crops as their primary food source [40], including cereals as a dietary staple (e.g., the maize porridge known as c*ongee*/*zhou*) in many communities. When cereal-based staples constitute a significant share of caloric intake, they provides an insufficient supply of selenium and other trace elements [41]. Notably, such diets are also characteristic of other high-EC regions, including East Africa (e.g., *ugali*/*nsima* made from maize) and South America.

A critical limitation of our analytical methodology is the lack of adjustment for covariates in our modelling, meaning that confounding cannot be ruled out. Two primary sources of potential confounding exist in this investigation. First, lifestyle-related EC risk factors, such as alcohol and tobacco consumption, may have influenced our findings. However, our sex-specific analyses partly alleviate this concern, as these exposures are more prevalent among males than females in this [42] and other settings. Second, confounding may have arisen from other environmental geochemical parameters expected to spatially correlate with selenium, such as soil pH, bedrock geology, and other trace elements, warranting future multivariate analyses. Finally, while this investigation is concerned with the link between selenium and ESCC, all EC subtypes combined are an acceptable proxy in this setting, where more than 90% of cases are of squamous cell histology [43].

In conclusion, we found that soil selenium concentrations were negatively associated with EC incidence. Our findings, notwithstanding the limitations discussed, support the hypothesis that soil selenium plays a role in EC geospatial distribution and aetiology in high incidence areas. Future analyses at different spatial scales, adjusting for additional geochemical variables, may offer further insights into the causal nature of this association. Individual-level observational studies could help investigate the influence of selenium and other beneficial micronutrients on local spatial variation in EC risk, such as case–control studies incorporating environmental sampling of soil used to grow subsistence crops at the household level.

## Data Availability

Requests for the data used in this study can be made to the corresponding authors.
